# Termination of Ventricular Tachycardia with  Antitachycardia Pacing after Ineffective Shock Therapy in an ICD Recipient with Hypertrophic Cardiomyopathy

**Published:** 2009-01-07

**Authors:** Panagiotis N Margos, Rolf Schomburg, Jorg Kynast, Ahmed A Khattab, Gert Richardt

**Affiliations:** Herz-Kreislauf-Zentrum, Segeberger Kliniken GmbH, Bad Segeberg-Germany

**Keywords:** Implantable Defibrillator, Antitachycardia Pacing, Hypertrophic Cardiomyopathy

## Abstract

Implantable Cardioverter Defibrillator (ICD) implantation is the only established therapy for primary or secondary prevention of sudden cardiac death in patients with Hypertrophic Cardiomyopathy (HCM). Ineffectiveness of shock therapy for the termination of potentially fatal ventricular arrhythmias in ICD recipients is rare in the presence of appropriate arrhythmia detection by the device. We report the case of a 48-year-old woman with HCM and a single chamber ICD, who received five inefficient high-energy (35 Joules) shocks for the termination of an appropriately detected episode of Ventricular Tachycardia (VT). The episode was safely terminated with a subsequent application of Antitachycardia Pacing (ATP) by the device. At the following ICD control, an acceptable defibrillation threshold was detected.

## Introduction

Implantable Cardioverter-Defibrillators (ICDs) can  terminate  ventricular  tachycardias (VTs) painlessly with antitachycardia pacing (ATP), especially              in episodes of spontaneous VT with cycle length (CL) >300 ms [1-3], while concerns   exist about efficacy and  safety of ATP in VT episodes with shorter CL (Fast VT, FVT) [2,3]. High-energy intracardiac shocks are almost always effective for the termination of VTs in the absence of ICD dysfunction [4].

We report a case of efficient ATP therapy for the termination of a FVT episode after five inefficient high-energy (35 Joules) intracardiac shocks in an ICD recipient with Hypertrophic Cardiomyopathy (HCM). The post-episode ICD control revealed  normal functional parameters of the device and acceptable defibrillation threshold test.

## Case

An 48-year-old female ICD recipient was admitted to our institution due to an episode of tachycardia, which was detected as VT and was followed by five consecutive  ineffective discharges of the device. The patient had a history of Hypertrophic-Obstructive Cardiomyopathy (HOCM) with transcoronary ablation of septal hypertrophy (TASH) 7 years ago and was receiving 240 mg of Verapamil daily. A single-chamber  (VVI)  ICD was implanted to the patient one year ago for secondary prevention after an episode of spontaneous sustained VT. [Single Chamber-ICD Medtronic™ Model: Maximo VR 7232, Singlecoil electrode 6931 Medtronic™; Parameter: VT 330ms (Therapy: Rx1: 3 sequences Burst 5 Impulses R-S1 Intervall 84%; Rx2: 3 sequences Ramp 5 Impulses R-S1 Interval 81%: Rx 3-6: cardioversion 35J), FVT 250ms (Therapy: Rx1: 1 sequence Burst 5 Impulses R-S1 Interval 88%: Rx 2-6: cardioversion), VF 280ms (Therapy 6 times DC-Shock 35J). SVT discrimination: Stability 30ms, Onset: 81%. Stimulation parameter: VVI 60 bpm ]. On transthoracic echocardiographic examination, the left ventricular systolic function was normal with  no detectable intraventricular gradient, while routine laboratory blood tests showed normal findings.

**Figure 1:**
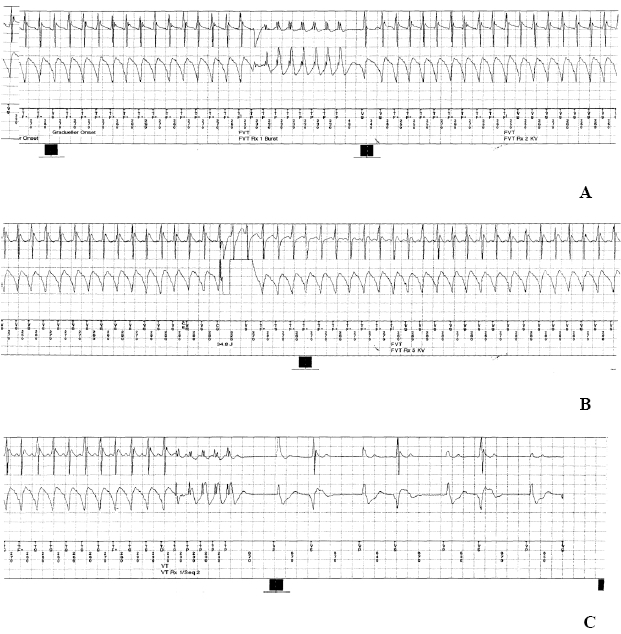
**A:** First ineffective ATP burst after ICD detection of FVT episode. **B:**First (among five) ineffective maximum-energy shock. **C:** Application of a new APT burst terminates the tachycardia.

A few days before admission, the patient was abruptly awaked  by consecutive discharges of the ICD. The following ICD control revealed an episode of an almost regular tachycardia with cycle length 330 to 270ms (spontaneously accelerated tachycardia), interpreted by the ICD as VT. The subsequent intervention of the device started with one ineffective ATP burst which was followed by five maximum-energy shocks (35 Joules), which were also ineffective. Finally, the tachycardia was successfully terminated  after  the application of a new ATP burst (Figure 1).

**Figure 2:**
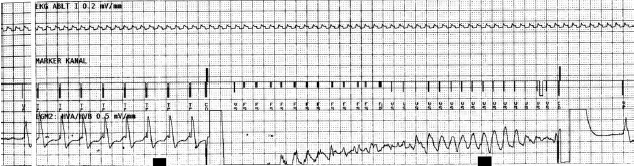
Successful testing of DC-shock-therapy after induction of VF by T-wave shock.

The rest findings of the ICD control regarding the functional  parameters of the can  and electrode were normal and the defibrillation threshold test was also acceptable, below 17 Joules (Figure 2). The patient's rest ECG revealed atrial fibrillation (of unknown onset) and pacing ventricular rhythm. Coronary angiography was performed showing normal coronary arteries, while left ventriculography confirmed the echocardiographic findings.

Regarding that the unusual outcome of ICD intervention (ineffective maximum-energy shocks followed by effective ATP burst) is compatible with inappropriate  ICD detection of a Supraventricular Tachycardia (SVT) as VT, the patient underwent Electrophysiological Study (EPS).  After the placement of two quadripolar electrodes in  right atrium and  right ventricle, an external defibrillation terminated atrial fibrillation and  also revealed the presence of Complete Heart Block (CHB) during sinus rhythm. Although the subsequent application of   pacing maneuvers did not produce any sustained tachycardia (Figure 3Aand B) we finally concluded that the clinical  episode was an appropriately detected VT, as the presence of CHB after cardioversion practically excludes the possibility of a prior  SVT episode with fast ventricular response.

Taking under consideration the absence of any detectable dysfunction of  the device (normal sensing and pacing function and, especially, acceptable defibrillation threshold) we suggested conservative treatment to the patient replacing verapamil with metoprolol plus amiodarone  and reprogramming the ICD therapy zones  in favor of ATP  versus shock therapy. Parameter: VT 330ms (Therapy: Rx1: 3 sequences Burst 5 Impulses R-S1 Interval 84%; Rx2: 3 sequences Ramp 5 Impulses R-S1 Intervall 81%: Rx3: 3 sequences Burst 5 Impulses R-S1 Interval 84%; 4-6 cardioversion 35J), FVT 270ms (Therapy: Rx1-3: 1 sequence Burst 5 impulses R-S1 Intervall 84%; Rx 4-6: 3 times cardioversion 35J), VF 280ms (Therapy: 6 times DC-Shock 35J). SVT discrimination: Off. Stimulation parameter: VVIR 60 - 120 bpm.

## Discussion

Ventricular tachycardia or fibrillation is usually the primary cause of sudden death  in patients with Hypertrophic Cardiomyopathy (HCM) and the only established therapy for the prevention of  sudden cardiac death in such patients  is the implantation of ICD [5,6]. Ventricular tachycardias  in patients with HCM may have focal origin (triggered activity, abnormal automaticity or microreentry circuit) while macroreentrant  mechanisms  are increasingly recognized in these patients, related with the presence of scar areas (subendocardial or intramural scars, revealed by contrast-enhanced MRI) [7] or apical aneurysm [8].

Previous reports have shown that ATP is  effective in terminating 80- 95%  of episodes of spontaneous VT with CL >300 ms [1-3]. Although VTs with shorter cycle length are usually treated with shocks  (ATP therapy has the risks of  tachycardia acceleration and syncope due to delay of definitive shock therapy) evidence support that  ATP can  safely terminate 40-80% of   FVTs as well [9,10], so as in current use of ICDs an ATP can precede the shock therapy, while the device is charging for shock delivery.

Ineffectiveness of shock therapy for the termination of detected episodes of VT or Ventricular Fibrillation (VF) in ICD recipients is  rare [4,11,12]. In these patients, false diagnosis of  VT or Ventricular Fibrillation (VF) not responding to ATP or shock therapy is due to ICD dysfunction or inappropriate detection of an SVT episode as VT, while real incessant VT or VF  is usually due to  acute myocardial ischemia and/or acute pump failure. Other possible explanations include antiarrhythmic drug toxicity (proarrhythmia), extreme electrolytic disorders and excessive alcohol  intake [11,12]. High defibrillation threshold has also been reported for  patients with Brugada syndrome [13].

The efficacy of ICD therapy in terminating VT or VF  in patients with HCM has been documented, although the myocardial thickness and disarray, which are characteristic for this disease, raise theoretical concerns about this [5,6]. Additionally, the reported incidence of inappropriate shock therapy in patients with HCM is comparable with the total incidence among ICD recipients [5,6].

In our case, our first aim was to examine the appropriateness of the ICD detection of VT  in comparison to the more reasonable (according  to the response to therapy) explanation of  inappropriate detection  of SVT. Regarding that single-chamber ICDs  perform only intraventricular recordings  that cannot reassure the accurate differential diagnosis between VT and regular SVT, the diagnosis of VT in our  patient was finally confirmed during the EPS.

Although there was no detectable apical aneurysm in  echocardiographic study and left ventriculography, the macroreentrant mechanism is still reasonable for the initiation and perpetuation of this VT episode, as the presence of subendocardial or intramural scar areas cannot be excluded (MRI was not performed). Moreover, Bundle Branch Reentry VT can be considered, as the patient has severe conduction system disease.  Regarding the possible focal origin of this VT episode, the spontaneous acceleration of  VT before ICD intervention  (warm-up phenomenon) in addition to the ineffectiveness of shock therapy and the final response to ATP therapy make abnormal automaticity more reasonable mechanism, while triggered activity and microreentry cannot be excluded as well.

The failure of five consecutive  maximum-energy ICD shocks to terminate the VT episode can not be easily explained in the absence of ICD dysfunction, excessive myocardial disease, proarrhythmia or  electrolytic disorders. Additionally,  VT was not inducible during the EPS and the reproducibility of this unusual outcome of  ICD therapy could not be examined. In any case, different mechanisms are responsible for the perpetuation of a VF episode (either primary or due to degeneration of a VT episode) than VT.  In our case, this aspect as well as the acceptable defibrillation threshold test, make unreasonable any concern about no response to shock therapy of a possible VF episode in the future.

According to the above, we applied a non-invasive therapeutic strategy for our patient, reforming the medical treatment and the ICD therapy zones. Radiofrequency ablation therapy has no significant role in patients with sustained VT due to HCM and is attempted  mainly in cases with macroreentrant VTs due to apical aneurysm [8]. Beta-blockers may increase the efficacy of ATP therapy, indicating a possible influence of autonomic tone in the maintenance of the VT [10]. The addition  of amiodarone to Beta-blocker might furthermore reduce the incidence of shock therapy in  patients with ICD, while the resultant  increase in the defibrillation threshold is small [14,15]. Among other antiarrhythmic drugs that may reduce the  incidence of ICD shocks, sotalol is the most reasonable alternative.

In conclusion, this is an unusual (to our knowledge the first reported) case of repeatedly ineffective maximum-energy  ICD  discharge for the termination of a FVT episode followed by effective ATP therapy,  in an ICD recipient with HCM, normal post-episode ICD control and acceptable defibrillation threshold. Concern about the efficacy of shock therapy in a possible future VF episode doesn't seem reasonable. Conservative therapy was suggested to the patient as there was no apparent role for any invasive therapeutic procedure.  Further investigation is needed about the mechanisms of initiation, perpetuation and termination of ventricular arrhythmias in patients with HCM.

## Figures and Tables

**Figure 3A:**
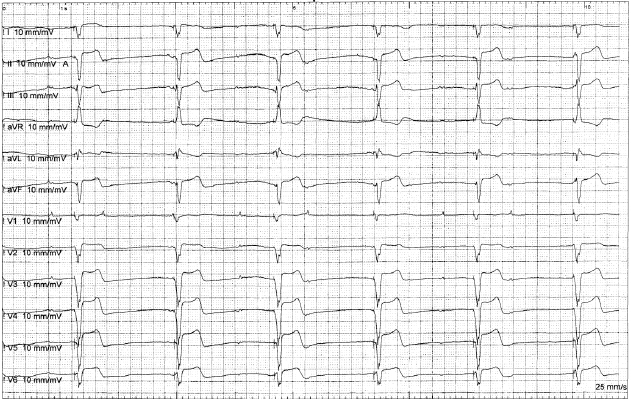
Complete Heart Block after external defibrillation for Atrial Fibrillation (Ventricular pacing at 40 bpm).

**Figure 3B:**
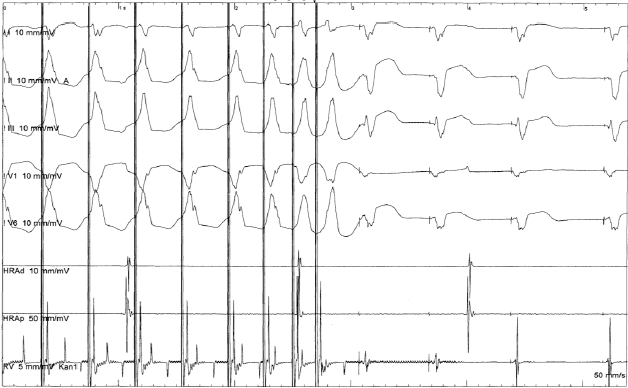
Ventricular stimulation for the induction of VT (RVOT pacing, three extrasystoles). Atrial and ventricular stimulation before and after i.v. administration of isoproterenol didn't produce any sustained tachycardia.
